# PANI–WO_3_·2H_2_O Nanocomposite: Phase Interaction and Evaluation of Electronic Properties by Combined Experimental Techniques and *Ab-Initio* Calculation

**DOI:** 10.3390/molecules27154905

**Published:** 2022-07-31

**Authors:** Adriano de Souza Carolino, Matheus Moraes Biondo, Ştefan Ţălu, Henrique Duarte da Fonseca Filho, Pedro Henrique Campelo, Jaqueline de Araújo Bezerra, Cicero Mota, Hidembergue Ordozgoith da Frota, Vanderlei Salvador Bagnato, Natalia Mayumi Inada, Edgar Aparecido Sanches

**Affiliations:** 1 Laboratory of Nanostructured Polymers (NANOPOL), Federal University of Amazonas (UFAM), Manaus 69067-005, AM, Brazil; adriano.asc.fis@gmail.com (A.d.S.C.); matheusbiondo@ufam.edu.br (M.M.B.); 2 Graduate Program in Physics (PPGFIS), Federal University of Amazonas (UFAM), Manaus 69067-005, AM, Brazil; hdffilho@ufam.edu.br (H.D.d.F.F.); hfrota@ufam.edu.br (H.O.d.F.); 3 The Directorate of Research, Development and Innovation Management (DMCDI), Technical University of Cluj-Napoca, 15 Constantin Daicoviciu St., 400020 Cluj-Napoca, Romania; 4 Laboratory of Synthesis of Nanomaterials and Nanoscopy (LSNN), Federal University of Amazonas (UFAM), Manaus 69067-005, AM, Brazil; 5 Department of Food Technology, Federal University of Viçosa (UFV), Viçosa 36570-900, MG, Brazil; pcampelo.felix@gmail.com; 6 Analytical Center, Federal Institute of Education, Science and Technology of Amazonas (IFAM), Manaus 69020-120, AM, Brazil; jaqueline.araujo@ifam.edu.br; 7 Department of Mathematics, Federal University of Amazonas (UFAM), Manaus 69067-005, AM, Brazil; mota@ufam.edu.br; 8 São Carlos Institute of Physics (IFSC), University of São Paulo (USP), São Carlos 13563-120, SP, Brazil; vander@ifsc.usp.br (V.S.B.); nataliainada@ifsc.usp.br (N.M.I.); 9 Hagler Institute for Advanced Studies, Texas A&M University, College Station, TX 77843-3572, USA

**Keywords:** nanocomposite, polyaniline, tungsten oxide, electronic properties, DFT calculation

## Abstract

The development of conjugated polymer-based nanocomposites by adding metallic particles into the polymerization medium allows the proposition of novel materials presenting improved electrical and optical properties. Polyaniline Emeraldine-salt form (ES–PANI) has been extensively studied due to its controllable electrical conductivity and oxidation states. On the other hand, tungsten oxide (WO_3_) and its di-hydrated phases, such as WO_3_·2H_2_O, have been reported as important materials in photocatalysis and sensors. Herein, the WO_3_·2H_2_O phase was directly obtained during the in-situ polymerization of aniline hydrochloride from metallic tungsten (W), allowing the formation of hybrid nanocomposites based on its full oxidation into WO_3_·2H_2_O. The developed ES–PANI–WO_3_·2H_2_O nanocomposites were successfully characterized using experimental techniques combined with Density Functional Theory (DFT). The formation of WO_3_·2H_2_O was clearly verified after two hours of synthesis (PW_2_ nanocomposite), allowing the confirmation of purely physical interaction between matrix and reinforcement. As a result, increased electrical conductivity was verified in the PW_2_ nanocomposite: the DFT calculations revealed a charge transfer from the *p*-orbitals of the polymeric phase to the *d*-orbitals of the oxide phase, resulting in higher conductivity when compared to the pure ES–PANI.

## 1. Introduction

The development of novel materials presenting simple methods of synthesis/processing has been increasingly considered in recent studies. The interest in nanocomposites is mainly due to their improved properties when compared to the individual phases [1,2]. Simple and fast methodology of synthesis has been proposed by polymerization reactions in the presence of inorganic particles. Moreover, the obtainment of specific inorganic reinforcement phases has also been reported as by-products of the in-situ polymerization [1,3].

Several inorganic reinforcements have been combined to Intrinsically Conducting Polymers (ICP) matrices to prepare novel nanocomposites [4,5]. As a result, some polymer properties are significantly improved. The incorporated inorganic reinforcements usually have high specific area, allowing better dispersion into the polymer matrix. Thus, important modifications in electrical, optical, thermal, morphological, and structural properties are related to the phase interactions of nanocomposites [1,6].

Polyaniline (PANI), one of the most studied ICP, is still a promising material for technological applications, as well as for the development of novel nanocomposites. The importance of PANI in several studies is mainly due to its ease of synthesis and doping, high molecular mass, and purity. Moreover, its doped form, known as Emeraldine-salt of polyaniline (ES–PANI), presents a wide range of electrical conductivity [7,8].

The combination of ES–PANI with inorganic particles has been widely reported in scientific literature. Usually, the resulting nanocomposites present enhanced electrical and thermal properties. The matrix-reinforcement interaction allows the evaluation of several phenomena resulting in modified crystal structure and morphology, charge transfers, electrostatic interactions, formation of new chemical bonds, and new electronic properties [9,10,11,12]. The scientific literature has reported polymeric matrix-based nanocomposites formed by ICP and inorganic particles presenting physical [13] or chemical [12] phase interactions. Significant improvement in electrical properties of nanocomposites was revealed by the incorporation of Al_2_O_3_ and CuO into the aniline polymerization reaction medium. However, there is lack of information on nanocomposites formed by ES–PANI and tungsten oxides, especially WO_3_·H_2_O and WO_3_·2H_2_O.

Combined experimental and theoretical data of nanocomposites formed by ES–PANI and WO_3_·2H_2_O (di-hydrated tungsten oxide, containing water molecules layered between the oxide molecular chains), to the best of our knowledge, has not been reported in scientific literature [14]. Tungsten oxides have been considered as promising materials, especially as reinforcement phases of conducting polymer-based nanocomposites. Their technological applications as photocatalysts and proton diffusion conductors have been reported elsewhere [15,16].

A novel synthesis mechanism to prepare a nanocomposite based on ES–PANI and WO_3_·2H_2_O is proposed herein. This di-hydrated oxide is usually obtained from Na_2_WO_4_·2H_2_O [11,17,18], which is incorporated into polyaniline to form a nanocomposite. In the present research, the obtainment of WO_3_·2H_2_O phase is proposed from the oxidation of metallic W simultaneously to the aniline polymerization. The phases formation was accompanied by different times of synthesis, allowing the evaluation of the developed hybrid nanocomposite by combined experimental techniques and theoretical calculation through the Density Functional Theory (DFT).

## 2. Results and Discussion

### 2.1. XRD Analysis

**Figure 1:**
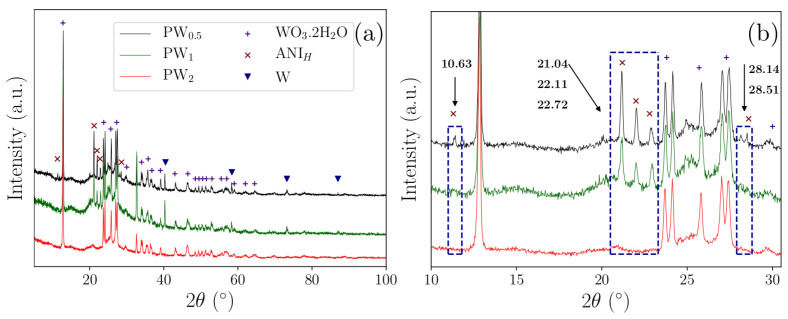
XRD patterns of PW_0.5_, PW_1_ and PW_2_ nanocomposites: (**a**) Diffraction peaks highlighting individual phases assigned to ES–PANI, aniline hydrochloride and WO_3_·2H_2_O, in addition to residual metallic W; (**b**) Angular region (in 2*θ*) from 10° to 30° for better visualization of the diffraction peaks.

XRD analysis allowed the obtainment of the diffraction patterns of the phases formed during the nanocomposite’s development (PW_0.5_, PW_1_ and PW_2_), as shown in Figure 1a. After 0.5 h and 1 h of synthesis (PW_0.5_ and PW_1_, respectively), the aniline polymerization reaction in the presence of O_2_, HCl, and ammonium persulfate (APS) resulted in the formation of individual phases assigned to ES–PANI, aniline hydrochloride and WO_3_·2H_2_O, in addition to residual metallic W (Equation (1)).

(1) $$ANI_{(}{{}_{L}}_{)}+W_{(}{{}_{S}}_{)}+O_{2}+H_{2}O\overset{HCl,\;APS}{\to }ES-PANI_{(}{{}_{S}}_{)}+ANI_{H}{{}_{(}}_{S}{}_{)}+WO_{3}•2H_{2}O+W_{(}{{}_{S}}_{)}$$

The semi-crystalline diffraction pattern of ES–PANI was not clearly observed in the nanocomposite forms. However, its most intense diffraction peaks were observed between 2*θ* = 20°–40°. As a semi-crystalline material, the contribution of the ES–PANI phase was observed and characterized by the non-crystalline halo located basically in the same angular region [19].

The formation of aniline hydrochloride phase was observed in PW_0.5_ and PW_1_ nanocomposites as a non-polymerized doped monomer. This phase presented diffraction peaks at 2*θ* = 10.6°, 21.0°, 22.1°, 22.7° and 28.1° [20]. Figure 1b shows the disappearance of this phase when the time of synthesis was increased for the formation of the PW_2_ nanocomposite. However, only by increasing the concentration of APS, and after 2 h of synthesis, the aniline hydrochloride monomers were fully polymerized to form the ES–PANI phase.

The metallic W phase was easily oxidized to tungsten trioxide (WO_3_). However, this phase was not observed in the prepared nanocomposites. Instead, a di-hydrated tungsten oxide (WO_3_·2H_2_O) resulted from the oxidation process of metallic W during the polymerization of aniline. Different tungsten oxides have been reported in scientific literature. The obtainment of WO_3_·2H_2_O suggested that the metallic W was oxidized by APS/O_2_ and interacted with water molecules from the solution. This phase was clearly identified [21], presenting diffraction peaks at 2*θ* = 12.8°, 23.7°, 24.1°, 27.0° and 27.4°, which corresponded to the planes (010), (001), (200), (011) and (210), respectively. Similarly, the metallic W phase was also identified in the PW_0.5_ and PW_1_ nanocomposites with diffraction peaks at 2*θ* = 40.3°, 58.3°, 73.2° and 87.0° [22]. After 2 h of synthesis, and using an increased concentration of APS, the metallic W phase was completely converted into WO_3_·2H_2_O (Figure 1b).

Changes in diffraction peak intensities were observed as a function of the time of synthesis. A decreased intensity of the diffraction peaks of aniline hydrochloride phase was observed as a function of time, and disappeared completely after 2 h (Figure 1b). No angular shifts in 2*θ* were observed, revealing that the crystalline structure and unit cells of all nanocomposite phases were maintained when the metallic W was converted to WO_3_·2H_2_O, as well as when the aniline hydrochloride monomers were polymerized. Then, after 2 h, the nanocomposite formed by ES–PANI and WO_3_·2H_2_O (PW_2_) was successfully obtained.

Nanocomposites formed by ES–PANI and tungsten oxides have been applied in several technological applications [14,15,23], and WO_3_ is the most reported form. The obtainment of this structure usually applies sodium tungstate di-hydrated (Na_2_WO_4_·2H_2_O), hydrochloric acid, oxalic acid, and deionized water. The system is then heated to remove water molecules [11,17,18]. However, in the methodology of preparation proposed herein, the WO_3_·2H_2_O phase was simultaneously obtained during the polymerization of aniline using metallic W as precursor. The XRD results showed that the WO_3_·2H_2_O phase was dependent both on the time of synthesis and APS concentration. This result indicated an alternative synthesis route to obtain PANI–WO_3_·2H_2_O nanocomposite without using Na_2_WO_4_·2H_2_O as a precursor material.

### 2.2. Structural Model of the Prepared Nanocomposites

The crystal structure of the aniline tetramer [24] was used as initial parameters consisting of four monomers located along the *z*-direction. Some calculations reported elsewhere [25] were also considered. The crystal structure of WO_3_·2H_2_O was based on a previous report [21] and allowed the obtainment of its atomic coordinates based on the insertion of water molecules. The correction for the Coulombian interaction (DFT+U) for strongly correlated systems was considered and allowed the obtainment of a more accurate model with good representation of the *gap* energy.

**Figure 2:**
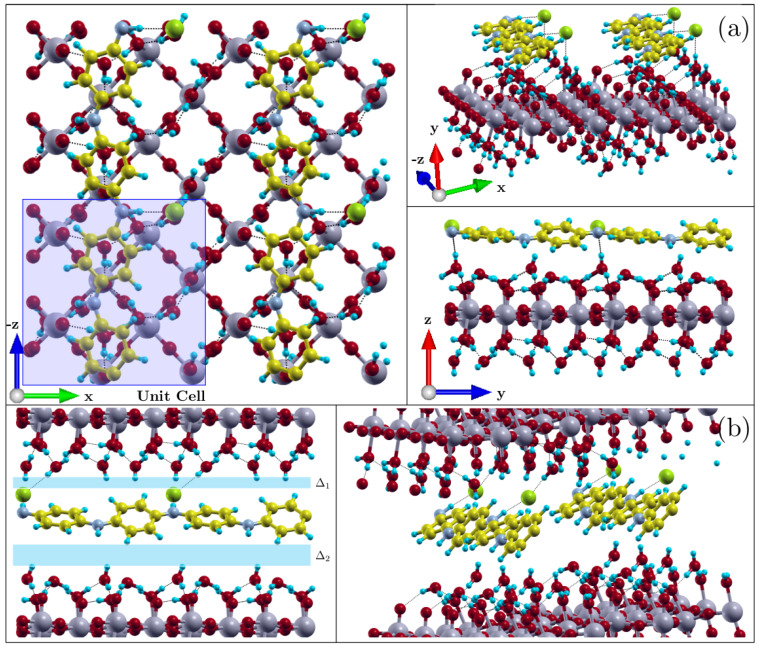
(**a**) Optimized structure of the periodically replicated PWO–ClS system (unit cell highlighted in blue). The polymeric phase was deposited on a WO_3_·2H_2_O layer and, after geometric optimization, presented a distance of 0.60 Å. No changes in their molecular structures were observed, only a rearrangement in their bond lengths. (**b**) Optimized structure of the periodically replicated PWO–ClB system. The polymer phase interacted with two layers of WO_3_·2H_2_O, presenting distances of Δ_1_ = 1.17 Å and Δ_2_ = 1.78 Å between the upper and bottom layers, respectively. No changes in their molecular structures were observed, only a rearrangement in their bond lengths in addition to the interaction between counter ions, polymer layer, and upper oxide layer.

From the DFT+U calculation, the structure of ES–PANI (doped with Cl^−^ counter ions) layered on a WO_3_·2H_2_O plate was obtained after geometric optimization. Two arrangements were chosen to calculate the electronic properties of the nanocomposite. The first one was based on a supercell considering a vacuum region of 15.55 Å between two adjacent layers with constant total energy, allowing the evaluation of the phase interactions along the *xz*-plane (Figure 2a). The second calculation was based on the bulk structural arrangement (Figure 2b). In addition, the same structures were analyzed as undoped forms, resulting in 4 models, labeled as PWO–ClS (doped system surface), PWO–NS (undoped system surface), PWO–ClB (doped system bulk) and PWO–NB (undoped system bulk).

**Figure 3:**
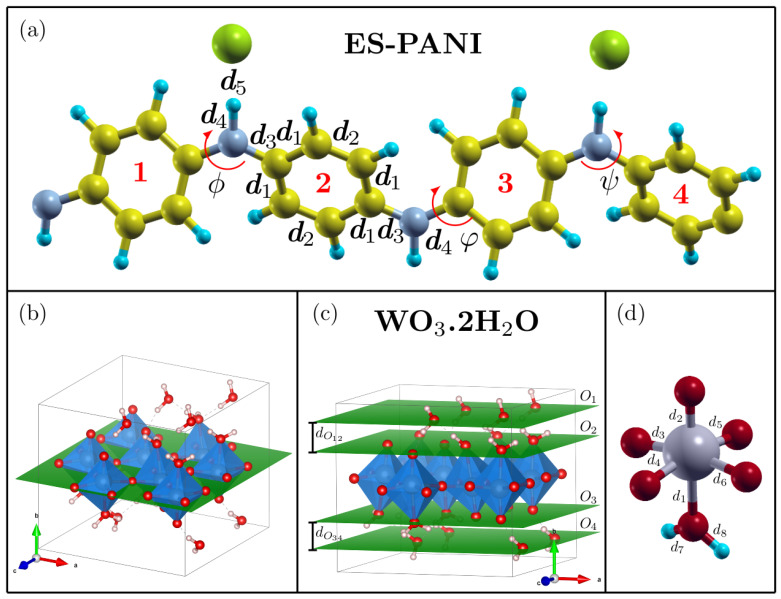
(**a**) Representation of the bond lengths and torsion angles between the rings of the optimized structure of ES–PANI phase interacting with the WO_3_·2H_2_O structure. (**b**) Planes formed by the central W and O atoms, (**c**) O_1_ and O_2_ planes formed by two water layers in the WO_3_·2H_2_O structure, and (**d**) Identification of the bond lengths of the WO_3_·2H_2_O unit described in Table 2.

The bond lengths (Table 1) of the nanocomposite phases were evaluated in all proposed systems. The bond lengths in polymer and oxide phases are shown in Figure 3a–d.

The binding energy of the PWO nanocomposite was calculated to access the stability of the phase interactions (Equation (2)):

(2) $$E_{bond}=E_{tc}-[E_{p}+E_{0}]$$

where *E_tc_* represents the total energy of the nanocomposite, and *E_p_* and *E_o_* represent the calculated energy of the individual phases.

The calculated binding energy of the PWO–ClS system was found around –0.109 eV, indicating a stability in the interaction between both phases, which occurred about 0.60 Å from the plane formed by the water molecules. Similarly, the calculated binding energy of the PWO–NS system was found around −0.103 eV, showing less stability in the interaction of phases when compared to the PWO–ClS nanocomposite.

The individual structures composing the PWO–ClB nanocomposite (*b* = 14.88 Å (Figure 2b)) preserved their configurations. No chemical bonds between phases were observed, and a negative binding energy of −0.090 eV was accessed, revealing stability. Similarly, the binding energy of the PWO–NB system was found around −0.237 eV, showing higher stability when compared to the other evaluated nanocomposites. Due to the Coulomb interactions, a difference in the length of the internal bonds of each phase was observed when compared to those of their isolated forms.

The polymer structure was rotated around 28° and attracted by 2 oxide layers upper and lower to the plane of the polymer chain. Hydrogen bonds were observed between the Cl and the H atoms of the upper layer of the water molecules. A plane in the *xz*-direction was considered at the threshold separating the phases in the PWO–ClB nanocomposite (two inside the unit cell and another one between two repeated cells along the *y*-direction). Then, a distance between the polymer phase and the upper and lower oxide phases was found to be ∆_1_ = 1.17 Å and ∆_2_ = 1.78 Å, respectively. Similarly, the structure of the undoped systems was obtained based on the same unit cell dimensions for surface area and bulk systems. Table 2 shows the distances between the WO_3_·2H_2_O layers and the polymer phase in all systems, as well as the distances between the hydrogen bonds and Cl (*d_Cl_*_−*H*_), and water molecules (*d_H_*_−*O*_).

The distance between the polymer phase and the oxide layer increased when the systems were relaxed to the bulk form. An interaction between the polymeric phase and both upper and bottom oxide layers was observed. Structural rearrangements were also observed by changes in hydrogen bond lengths from 1.36 Å to 1.89 Å, and from 1.36 Å to 2.02 Å in the doped and undoped structures, respectively.

### 2.3. FTIR Analysis

**Figure 4:**
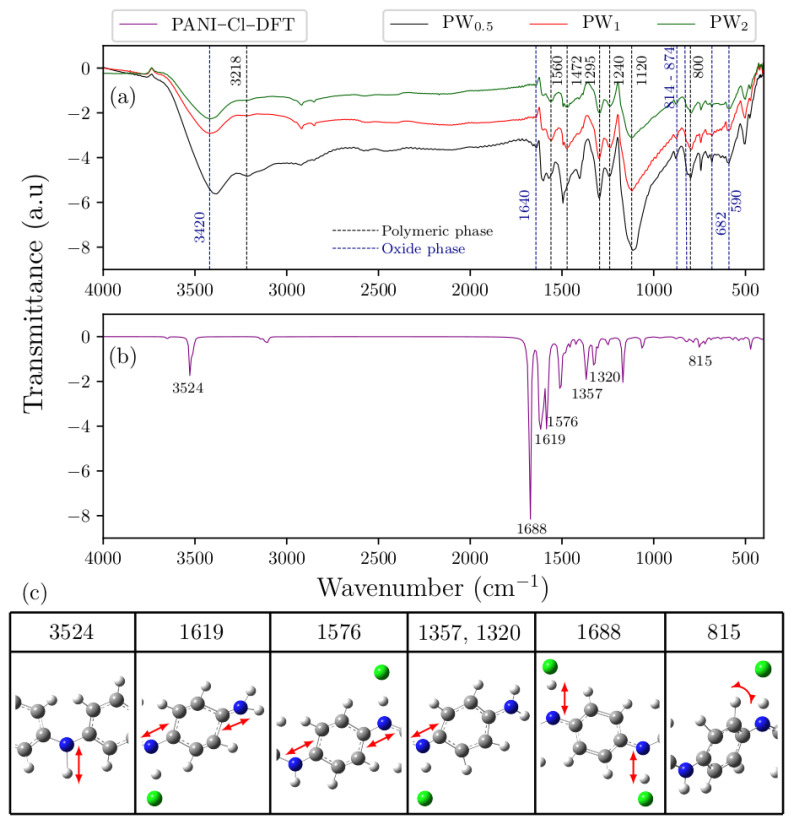
(**a**) Spectra of the *as*-synthesized PW_0.5_, PW_1_ and PW_2_ nanocomposites showing the bands corresponding to the main vibrational modes. The bands resulting from the stretching and deformations of the polymeric phase were clearly identified; (**b**) Theoretical spectrum of ES–PANI and (**c**) Observed vibrational modes.

Figure 4a,b shows the experimental FTIR spectra of PW_0.5_, PW_1_ and PW_2_ nanocomposites, as well as the DFT–based spectrum of ES–PANI polymer phase. The identified vibrational modes are highlighted in Figure 4c.

The ES–PANI polymer phase spectrum presented 7 main absorption bands at 3218 cm^−1^, 1560 cm^−1^, 1472 cm^−1^, 1295 cm^−1^, 1240 cm^−1^, 1120 cm^−1^ and 800 cm^−1^. The band located at 3218 cm^−1^ was assigned to the symmetric stretching of the N–H bond [26]. This band also showed a redshift in the theoretical spectrum to 3524 cm^−1^. The stretching of the quinoid and benzenoid structures were observed, respectively, at 1560 cm^−1^ and 1472 cm^−1^, allowing the characterization of the main molecular structure of ES–PANI. These bands presented a redshift, respectively, to 1619 cm^−1^ and 1576 cm^−1^ in the theoretical spectrum [27]. The absorptions at 1295 cm^−1^, 1240 cm^−1^ and 1120 cm^−1^ were assigned to the stretching of the C–N^+^ bond of the bipolaronic structure, and to the N–H^+^ bond from the delocalized *π*–electrons due to the protonation process [28]. The bands related to the C–N^+^ stretching were observed at 1357 cm^−1^ and 1320 cm^−1^ in the theoretical spectrum. However, the band related to the N–H^+^ stretching showed higher intensity at 1688 cm^−1^. Interestingly, with increasing the time of synthesis, the intensity of the bands in the range from 1295 cm^−1^ to 1120 cm^−1^ was decreased. The out–of–plane deformation of the C–H bonds of benzenoid rings [13] was observed at 800 cm^−1^ and at 815 cm^−1^, respectively, in the experimental and theoretical spectra.

The ratio between the areas of the quinoid and benzenoid (Q/B) structures (1560 cm^−1^ and 1462 cm^−1^, respectively) was useful to estimate the doping level of ES–PANI as a function of the time of synthesis [29]. The Q/B ratio was found to be 0.89 in all nanocomposites, suggesting that the PWO nanocomposite counter ion/chain distance observed in Table 1 did not result in polymer deprotonation when compared to the distances of the isolated systems.

The doping of polyaniline occurs through the interaction of counter ions and polymer chain, and the doped state occurs in a proportion of quinoid and benzenoid structures (related to the structural defects from these interactions). The distance between polymer and counter ions may reveal a possible deprotonation, returning an insulating state polymer. However, this fact was improbable because the Q/B ratio was greater than 50%, confirming the conductive behavior of the polymer.

**Figure 5:**
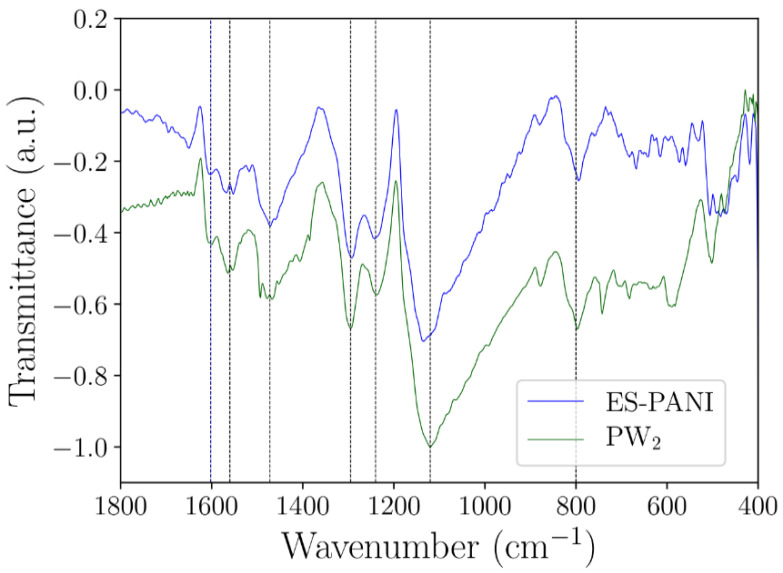
FTIR spectra of ES–PANI and PW_2_ nanocomposite.

Figure 5 shows the comparison between the ES–PANI and PW_2_ spectra. No peak shift (nor new absorption bands) was observed after the nanocomposite formation, pointing to a physical interaction between phases. Similar results were reported in [13], showing the in-situ incorporation of aluminum oxide into the aniline polymerization medium. The resulting nanocomposite presented enhanced electrical conductivity and electrostatic interaction between phases, with no shift or new absorption FTIR bands.

Despite presenting a physical interaction, some nanocomposites based on polyaniline and inorganic particles can also present chemical interaction. The nanocomposite formed by polyaniline and copper oxide [12] showed important absorption shifts on the FTIR spectrum. The chemical interaction between phases was also confirmed by the authors through DFT+U calculations.

Our results showed that all identified absorptions (Table 3) were maintained when the time of synthesis was increased. No intensity variations in the absorption bands related to the protonation of ES–PANI were observed, revealing no deprotonation of the polymer chains in the nanocomposite form.

The absorptions at 3420 cm^−1^, 1640 cm^−1^, 874 cm^−1^, 814 cm^−1^, 682 cm^−1^ and 590 cm^−1^ were observed in the WO_3_·2H_2_O spectrum. The absorptions at 874 cm^−1^ and 814 cm^−1^ were assigned to the O–W–O antisymmetric stretching [18], while the absorptions at 682 cm^−1^ and 590 cm^−1^ corresponded to the O–W–O symmetric stretching. The band at 1640 cm^−1^ was attributed to the in-plane O–H angular deformation [11], and the absorption at 3420 cm^−1^ was assigned to the O–H stretching from both moisture and water molecules of WO_3_·2H_2_O [30].

### 2.4. UV-VIS Analysis

**Figure 6:**
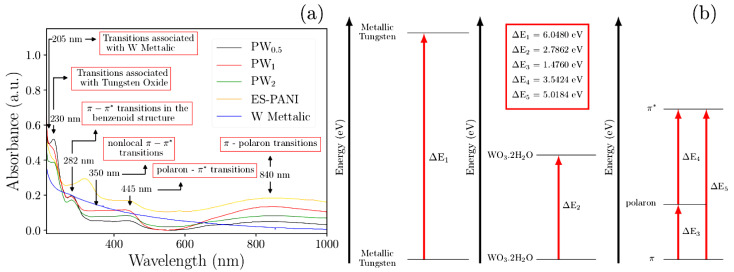
(**a**) UV-VIS spectra of the *as*-synthesized nanocomposites PW_0.5_, PW_1_ and PW_2_ showing the main polaronic state transitions, as well as the peaks related to the metallic W and WO_3_·2H_2_O phases. (**b**) Schematic representation of the electronic transitions between phases.

UV-VIS analysis was useful to investigate the main electronic transitions of the nanocomposites and also to evaluate the influence of WO_3_·2H_2_O in the polymer structure. The UV-VIS spectra of all systems are shown in Figure 6.

A number of 6 absorption bands resulted from the electronic transitions in all systems. The first absorption at 205 nm observed in the nanocomposite’s spectra was attributed to the W transitions, and a similar transition was also observed in the pure W spectra with a small blueshift.

Characteristic peaks from the polyaniline transitions were observed at 230 nm. The first one was attributed to the π–π* transitions of the benzenoid structure of the polymer chain, followed by the nonlocal π–π* transitions at 282 nm [31]. Absorptions from the transitions of the oxidized W was observed from 350 nm to 445 nm, with contribution from both WO_3_·2H_2_O and the polymeric phase [14,32]. Similar absorption of the polaron–π* transition was reported [31]. The absorption at 840 nm was attributed to *π–*polaron transitions associated with the polymer doping process [31].

The *π-*polaron transitions occurred because, when doped, polymers such as PANI and its derivatives present new energy states located within the gap and close to the lowest energy state (HOMO), which contain a single unpaired electron. The energy level associated with the polaron represents a destabilized bonding orbital and, therefore, presents higher energy than that of HOMO, allowing the π-polaron and polaron-π* transitions, responsible for the conductive behavior of ES–PANI.

All absorptions highlighted in Figure 6 are listed in Table 4. The lower energy electronic transitions were associated with the polymer as well as its doped form, which occurred due to the creation of polaronic states.

### 2.5. Complex Impedance Spectroscopy

**Figure 7:**
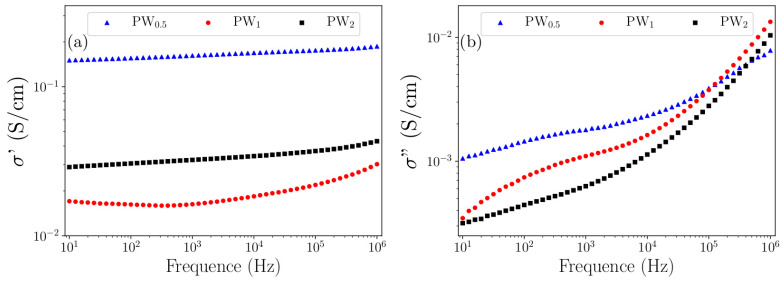
(**a**) Real and (**b**) Imaginary complex conductivity spectrum of nanocomposites.

The electrical conductivity of the prepared nanocomposites was analyzed by Complex Impedance Spectroscopy. Figure 7 shows (a) the dependence of the real and (b) imaginary parts of the complex conductivity as a function of frequency (Equations (3) and (4)):

(3) $$\sigma^{\prime }=\omega \cdot \varepsilon_{0}\cdot \varepsilon^{\prime •}$$

(4) $$\sigma^{\prime •}=\omega \cdot \varepsilon_{0}\cdot \varepsilon^{\prime }$$

where $\varepsilon^{\prime }$ and $\varepsilon^{\prime •}$ represent, respectively, the real and imaginary dielectric permittivity calculated from the complex impedance Z (Equations (5) and (6)):

(5) $$\varepsilon^{\prime }(\omega )=\frac{-Z^{\prime }}{\omega \cdot C\cdot [{\left(Z^{\prime }\right)}^{2}+{\left(Z^{\prime •}\right)}^{2}]}$$

(6) $$\varepsilon^{\prime •}(\omega )=\frac{-Z^{\prime •}}{\omega \cdot C\cdot [{\left(Z^{\prime }\right)}^{2}+{\left(Z^{\prime •}\right)}^{2}]}$$

The real part represents the conduction in phase with the applied electric field, while the imaginary part presents the out-of-phase conduction. It can be observed from the plot of the real part that PW_0.5_ and PW_2_ did not present significant changes in the electrical current when the frequency was increased, showing for both nanocomposites a *dc* conductivity. However, PW_1_ presented an increase in electrical conductivity at high frequencies. This behavior was assumed to accord with the relation known as Jonscher universal power law, where the electrical conductivity is independent of the frequency when n = 0, and dependent when n > 0. This result is supported by the fact that at low frequencies the disordered regions acted with high resistance, resulting only in a constant conductivity. However, at high frequencies the rate of hopping between the conductive islands and/or between phases increased the conductivity.

It was not possible to assess whether the conductivity maxima showed relaxation peaks resulting from the electrical conduction by hopping due to the applied frequency range in the PW_1_ imaginary part (around 10^2^ Hz). Thus, we suggested a combined electrical conduction in the nanocomposite form due to the doping characteristics of the polymer, the conduction of the metal phase, the type of ionic structure of the oxide phase, as well as the presence of water in the hydrated structure. This conduction is mostly *dc* from charge carriers generated through the charge defects of ES–PANI and from the free charge carriers coming from the remaining metallic W in PW_0.5_ and PW_1_. However, the electrical conduction is based on hopping between the interfaces when the energy is sufficient to break the potential barrier.

Table 5 presents the values of electrical conductivity of the prepared nanocomposites. PW_0.5_ reached 1.4 × 10^−1^ S/cm. For PW_1_ and PW_2_ the electrical conductivity values decreased by one order of magnitude, reaching 1.6 × 10^−2^ and 2.9 × 10^−2^ S/cm, respectively.

The XRD results showed previously that the metal phase was gradually converted to WO_3_·2H_2_O after 2 h of synthesis. As a result, a decreased electronic mobility was verified and assigned to the reduced free charges from the metallic W. This fact was due to its conversion to WO_3_·2H_2_O. Despite the interactions between WO_3_·2H_2_O and counter ions, the electrical conductivity of the nanocomposites was maintained, suggesting that the distancing of the counter ions observed in the theoretical PWO models in relation to ES–PANI did not result in deprotonation. Compared to the experimental conductivity data of the doped polymer phase, interaction between phases in PW_0.5_, PW_1_ and PW_2_ was observed, resulting in enhanced conductivity values when compared to the pure ES–PANI, as well as showing charge transfer that improved the electronic mobility.

### 2.6. Band Structure and Density of States (PDOS)

The energy band structures were calculated in the reciprocal space along (Z, Γ, Z) for the polymer system, (Γ, X, U, Z, Γ, S, Z) for the WO_3_·2H_2_O system and for all nanocomposites using (Γ, X, U, Z, Γ, S, Z), where (Z, Γ) corresponds to the polymeric chain growth direction of ES–PANI. For all systems, the Fermi energy was adopted as reference for the origin.

**Figure 8:**
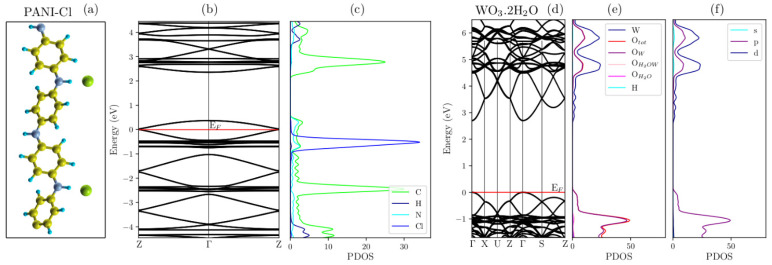
(**a**) Polymer system, (**b**) Its respective band structure and (**c**) PDOS for each atom. The system presented conducting behavior due to the half-filled energy bands. The PDOS confirmed the major contribution of the counter ions at the Fermi level. (**d**) Band structure, (**e**) PDOS for atom and (**f**) PDOS for orbital of the WO_3_·2H_2_O system using the Hubbard parameter, which influenced on the *gap* energy of 2.60 eV (similar to that of experimental results). The valence and conduction bands presented greater contribution of the *p* and *d*-orbitals from O and W atoms, respectively.

Figure 8a–c shows that the PANI–Cl system presented conductive material behavior, where the HOMO band was partially filled. This fact was due to the new energy states created by the addition of counter ions into the undoped PANI, allowing the transfer of electrons to lower energy states [25]. As a result, the *gap* energy was reduced from 2.00 eV (undoped PANI form [25]) to 0.41 eV (ES–PANI form).

The PDOS projected by atoms facilitated access of the major contribution of Cl atoms, followed by C and N (largest contribution in the LUMO band), as well as the lowest contribution of H atoms. The maximum of the PDOS of the Cl atoms occurred at approximately −0.15 eV below the last occupied state of the HOMO band. Then, the electrons from the higher energy states decayed to the energy states generated by the Cl atoms. For this reason, a charge transfer was allowed between polymer chains and counter ions.

Figure 8d–f shows the band structure and PDOS projected by atoms and orbitals for the WO_3_·2H_2_O system. The insertion of the Hubbard correction provided an excellent result for the *gap* energy of around 2.60 eV. Experimental *gap* energy of WO_3_·2H_2_O and WO_3_·H_2_O obtained by UV-VIS using the Wood-Tauc method were reported between 2.00 eV and 2.40 eV [14].

Figure 8e shows the distributed PDOS of the O atoms into 4 groups: O_tot_ represents the total electron density of oxygen, O_W_ is the density of oxygens in the *xz*–plane, O_H2OW_ represents the H_2_O molecules interacting with the W atoms in the *y*-axis, and O_H2O_ is the water molecules forming the upper and bottom layers. The O atoms in the *xz*–plane contributed significantly to the formation of the valence band. Marginal contribution of the O atoms from water, and perpendicular to the *xz*–plane, was observed. The PDOS of the O atoms was mainly related to the *p*–orbitals, as shown in Figure 8f. The major contribution in the conduction band was related to the W atoms, followed by O atoms in the *xz*–plane (where the *p* and *d*–orbitals present higher electron density). The electrons from the *d*–orbitals of the W atoms migrated to the *p*–orbitals of the unoccupied O atoms, so the *d*–orbitals did not contribute significantly in the valence band.

**Figure 9:**
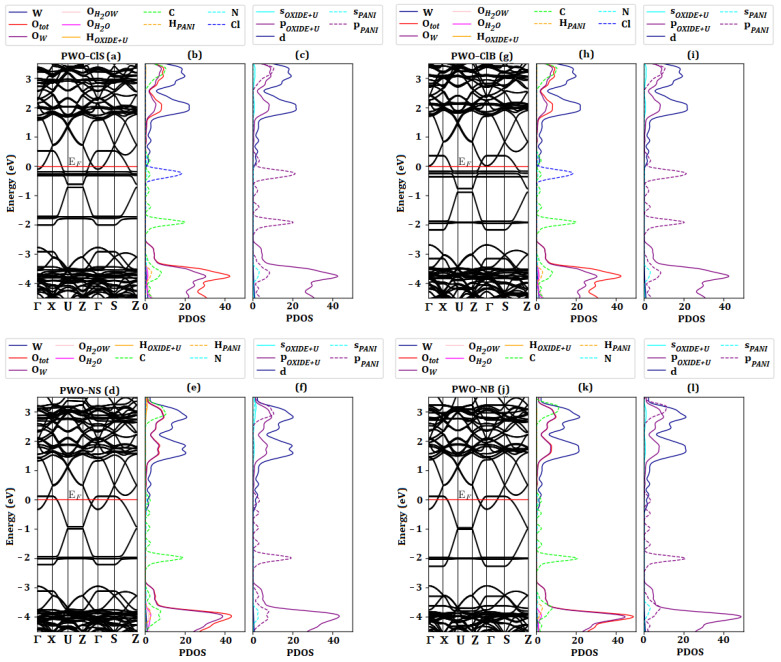
(**a**,**d**) Band structure, (**b**,**e**) PDOS for atoms and (**c**,**f**) PDOS for orbitals of PWO–ClS and PWO–NS systems. The counter ions caused a marginal energy difference in the Fermi level, shifting the position of the highest occupied state. The PDOS showed that the *p*–orbitals of the Cl and C atoms of the doped and undoped polymer phases, respectively, were responsible for promoting the electrons to the *d*–orbitals of the W atom. (**g**,**j**) Band structure, (**h**,**k**) PDOS for atoms and (**i**,**l**) PDOS for orbitals of PWO–ClB and PWO–NB systems. The counter ions caused a marginal energy difference in the Fermi level, shifting the position of the highest occupied state. This change was also attributed to the variation in the unit cell parameters. The PDOS showed that the *p*–orbitals of the Cl and C atoms of the doped and undoped polymer phases, respectively, were responsible for promoting the electrons to the *d*–orbitals of the W atom.

Figure 9a,d,g,j shows the energy band structure of all nanocomposites. The energy bands corresponding to the polymer phase were observed exactly in the region of the *gap* energy corresponding to the WO_3_·2H_2_O phase. All systems were similar, except for the states from the Cl atoms, and besides the difference in the Fermi level caused by the variation of the unit cell. In the doped systems the last occupied valence band state of WO_3_·2H_2_O was now at the conduction band limit because the polymer phase had higher energy filled levels. Thus, after excitement, the electrons from the polymer phase migrated to the higher unoccupied energy states of WO_3_·2H_2_O. Comparing the systems of PWO–ClS and PWO–ClB, a small difference in the Fermi energy was observed and related to the unit cell bulk change (∼−0.15 eV). The energy difference in the undoped systems was similar. On the other hand, the doped systems were influenced by counter ions, increasing the energy of the valence band of the WO_3_·2H_2_O phase.

Figure 9 b,c,e,f,h,i,k and l show the PDOS for atoms and for orbitals of both surface and bulk systems. The O and W atoms presented the major contribution in the valence and conduction bands. However, the largest contribution in the *gap* energy of the WO_3_·2H_2_O phase was related to the C, N and Cl (in doped systems, ES–PANI) atoms. The evaluation of the PDOS for orbitals revealed that the *p*–orbitals of the polymer phase were responsible for promoting the electrons to the *d*–orbitals of the W atoms (charge transfer between phases). For this reason, the polymer phase acted as a bridge in the *gap* energy of the WO_3_·2H_2_O phase in the nanocomposite forms, behaving as a conducting material. Even with the absence of the Cl atom, the undoped nanocomposite system exhibited the same characteristics as that of the doped one. The interactions between the nanocomposite phases created charge transfers from the *p*–orbitals of the polymer to the *d*–orbitals of the oxide phase, increasing the electronic mobility. Thus, the contribution of the C and N orbitals of the polymer phase, as well as the W and O orbitals of the oxide phase increased the PDOS of the nanocomposites, improving its electrical conductivity.

### 2.7. Charge Density

The Lowdin charge was adopted as the charge distribution parameter in order to analyze the charge flow in nanocomposites. Table 6 shows the Lowdin charge variation for PWO–ClS and PWO–NS systems. We represented Δ*C_Lowdin_* (PANI–Cl) here as the charge difference between the PWO–ClS nanocomposite and the PANI–Cl doped polymer, and Δ*C_Lowdin_* (WO_3_·2H_2_O) as the charge difference between the PWO–ClS nanocomposite and the WO_3_·2H_2_O phase. A decreased charge distribution of PANI–Cl of about 0.2459 was observed in relation to the nanocomposite form, and an equivalent increase of +0.2636 was observed in the WO_3_·2H_2_O phase. A difference in charge loss/gain between phases was also observed, which infringed the principle of charge conservation. This fact was due to imprecision in the bulk region regarding the position of each atom, resulting in some imprecision in the charge distribution calculation. However, our results revealed a satisfactory understanding of the charge mobility.

Similarly, the Δ*C_Lowdin_* (PANI) was described as the charge difference between the PWO–NS nanocomposite and the undoped polymer, and the Δ*C_Lowdin_* (WO_3_·2H_2_O) was assigned as the charge difference between the PWO–NS nanocomposite and the WO_3_·2H_2_O phase. A loss in charge distribution in the polymer phase of about −0.4961 (relative to the formed nanocomposite) was observed. On the other hand, an equivalent increase of +0.5226 was revealed in the WO_3_·2H_2_O phase. These values were twice those of the charge transfer between the doped state and the oxide phase.

**Figure 10:**
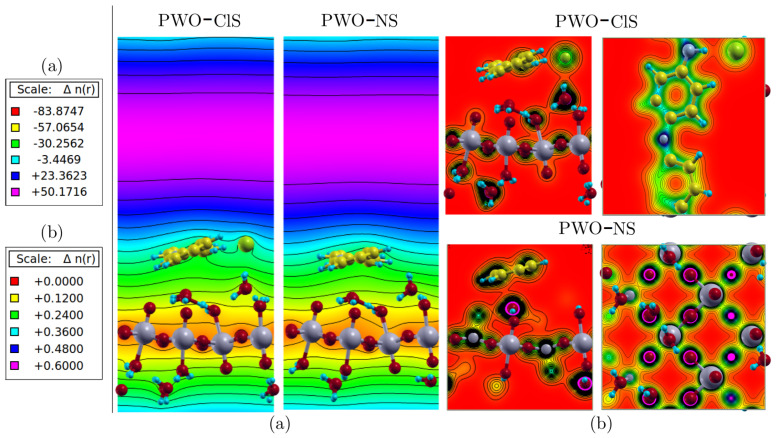
Ionic potential map and electric charge density on surface of the nanocomposite systems. (**a**) The scale of the ionic potential ranged from −83.87 (red) to +50.17 (purple) in a plane perpendicular to the growth of the polymer chain and (**b**) The scale of charge density ranging from 0 (red) to 0.6 (purple) in perpendicular and parallel directions to the *z*-axis, in which a higher distribution of charges was observed in the more electronegative atoms.

Figure 10 represents (a) the local ion potential map and (b) the charge distribution at the plane normal to the polymer chain growth, showing the electronic interactions between phases. The surface systems showed attractive ionic interactions between polyaniline and the WO_3_·2H_2_O surface. Higher electronic charge density in the O atom was revealed in the layer where polar W–O bonds along the *xz*–plane was observed. The Cl atom caused a marginal distortion in ionic potential distribution when compared to the undoped structure.

The density in both systems showed well-localized behavior, as shown in Figure 10a, and the highest density around the O atoms, followed by the C atoms along the polymer chain. However, intermediate regions of charge density were observed between the polymer phase and the Cl atom, as well as between the Cl atom and water molecules. In addition, an intermediate region between the water molecules and the oxygen atoms of the WO_3_·2H_2_O phase was also observed, as well as between the polymer chain and the WO_3_·2H_2_O phase. Based on color scale, as in Figure 10b, these regions presented low electron density. In the case of doped conducting polymers by protonation, it is known that the counter ions effectively participate in the electronic conduction, contributing to the electron neutrality of the polymer chain. Then, the electronic conduction occurs by intra- and inter-chain mechanisms, in addition to the hopping between the conducting islands formed by the crystalline regions.

The results observed in Figure 10 corroborate the experimental data observed by FTIR. The interaction occurring between polymer chains and WO_3_·2H_2_O phase was clearly electrostatic. The value of the attractive potential around −30.0 (atomic unit) is highlighted in green. This type of interaction resulted in the peak positions of the absorption bands in the FTIR spectra (Figure 5), since no band shifts/new bands were observed.

### 2.8. Transmittance and Electrical Current

**Figure 11:**
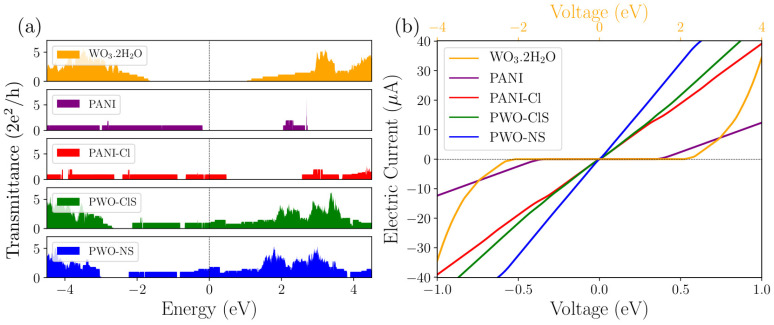
(**a**) Quantum transmittance spectrum of the proposed systems as a function of energy, considering EF = 0. The WO_3_·2H_2_O and PANI models showed *gap* energy around the Fermi level of 2.6 eV and 2.2 eV, respectively. The other models presented conducting behavior in terms of electronic charge transport. (**b**) Electric current curves as a function of applied voltage for all proposed systems. The isolated systems WO_3_·2H_2_O and PANI showed null electrical current in the ranges from 0.00 eV to 2.00 eV and from 0.00 eV to 0.37 eV, respectively, behaving as insulating materials. The doped PANI–Cl and the nanocomposite systems presented electrical current varying linearly as a function of the applied voltage, behaving as a metallic material.

Quantum transmittance and electric current as a function of voltage was calculated using the *Want* package [33] implemented in the Quantum Espresso software [34]. Figure 11a shows the plot of the quantum transmittance (2·e^2^/h) of the proposed systems as a function of energy (eV), considering the EF = 0.0.

All systems but PANI presented transmittance around the Fermi level, with no *gap* energy (as seen in the band structure). Thus, PANI–Cl, PWO–ClS and PWO–NS nanocomposites behaved as conductors in terms of electronic charge transport. A region of *gap* energy of approximately 2.2 eV around the Fermi level emerged in the PANI system, resulting in zero transmittance from −0.2 eV to 2.0 eV. For this reason, we suggest that PANI behaved as an insulating material, since it was the representation of the undoped form (leucoemeraldine).

Similarly, the WO_3_·2H_2_O system showed a *gap* energy of 2.6 eV around the Fermi level, with null transmittance from −1.6 eV to 1.0 eV. The transmittance spectra of the nanocomposites were similar, with a low difference in the Fermi level region resulting from the low influence of the Cl atom on the electronic conduction. The transmittance contributions of each phase were clear as they were quite characteristic when compared to their isolated states. Although PANI and WO_3_·2H_2_O present relatively large *gap* energy, the formation of PWO–NS and PWO–ClS nanocomposites exhibited conducting behavior, showing that the polymeric phase could act as an electronic bridge to reduce the *gap* energy of the oxide phase.

Figure 11b shows the curves of electrical current (A) as a function of applied voltage (eV) for all proposed systems. The IxV curve of the WO_3_·2H_2_O system presented non-ohmic behavior from 0.0 eV to 2.0 eV. For higher voltage values the electrical current increased exponentially. The WO_3_·2H_2_O phase represented an insulating material with no electrical conduction at low voltages. Similarly, the undoped polymer phase presented electrical current from 0.0 eV to 0.37 eV. The electrical current increased linearly from this voltage value. The null energy range of the polymer phase was smaller due to its narrower *gap* energy when compared to that of the oxide phase. Thus, it conducted electrical current over a smaller voltage range.

The ES–PANI and nanocomposite systems presented a characteristic curve of conducting materials, where the electrical current changed linearly with the applied voltage (typical ohmic behavior). A good approximation of the electronic properties was observed between the system formed only by PANI and WO_3_·2H_2_O (PWO) and the experimental PW system. Then, the final product ES–PANI–WO_3_·2H_2_O, which was prepared experimentally, presented enhanced conduction properties, and the PW_2_ nanocomposite exhibited higher conductivity than that of the individual phases.

### 2.9. Morphological Analysis

**Figure 12:**
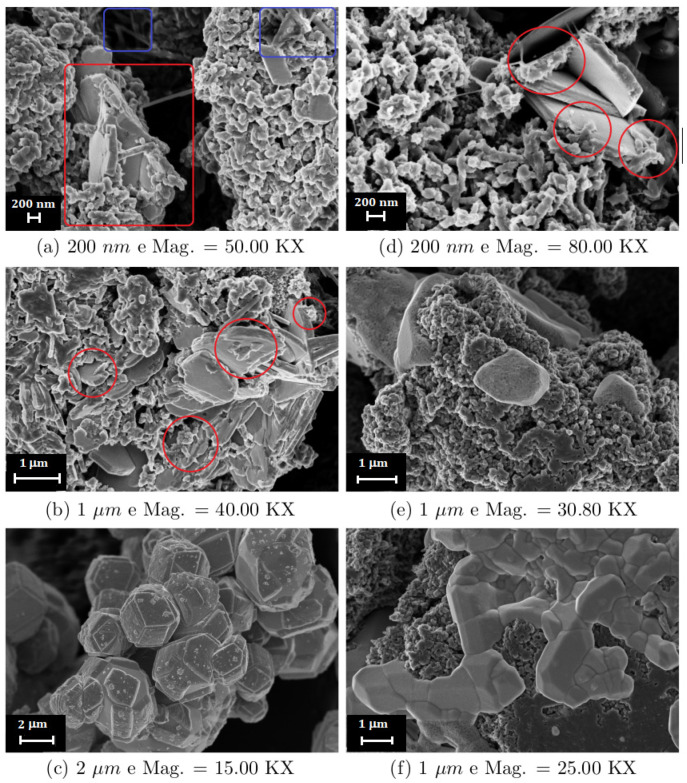
(**a**) Microplate morphology of the WO_3_·2H_2_O phase coexisting with ES–PANI and aniline hydrochloride (highlighted in blue); (**b**) Physical interaction between ES–PANI and WO_3_·2H_2_O phases (highlighted in red); (**c**) Regular morphology of metallic W; (**d**–**f**) PW_2_ nanocomposite morphology showing the physical interaction between WO_3_·2H_2_O microplates and ES–PANI fibers.

The morphology of the prepared PW_0.5_ and PW_2_ nanocomposites was evaluated by SEM images. The PW_0.5_ nanocomposite (Figure 12) clearly showed a morphology assigned to the polymeric phase (ES–PANI), constituted mainly of nanofibers [12,35]. Microplates of different sizes and thicknesses formed by the WO_3_·2H_2_O phase were also observed, as shown in Figure 12a,b [14]. The regions where ES–PANI was deposited on the WO_3_·2H_2_O microplates are highlighted in red, revealing a contact surface. The presence of nano-sticks morphology assigned to the aniline hydrochloride [19] phase is highlighted in blue in Figure 12a. In addition, the presence of the remaining metallic tungsten was also observed in Figure 12c, corroborating the results from XRD analysis. The regularity of the morphology of the metallic W was also noted, due to the high symmetry of its crystal structure.

The PW_2_ nanocomposite morphology Figure 12d–f was similar to that of PW_0.5_, showing predominantly the polymeric phase. However, the morphology of pure metallic W and aniline hydrochloride was not observed. The morphology assigned to the aniline hydrochloride in the PW_0.5_ nanocomposite was not found in the PW_2_ sample, corroborating the XRD results. The formation of WO_3_·2H_2_O [14] was clearly evidenced, showing that the metallic W was oxidized. The phase interaction between WO_3_·2H_2_O and polyaniline was clearly observed, corroborating our previous results pointing to a purely electrostatic physical interaction. This observed physical interaction may also be related to the increased electrical conductivity of the PW_2_ nanocomposite when compared to the pure ES–PANI, possibly due to new electronic conduction paths created in the nanocomposite material.

## 3. Experimental

### 3.1. Nanocomposites Preparation

The nanocomposite preparation was performed based on previous reports with some modification [36]. Two solutions were prepared. Solution I: An amount of 20 mL of aniline (ANI) was added to 500 mL of 1 M hydrochloric acid (HCl). An amount of 4.67 g of metallic W was added to 5 mL of distilled water under constant magnetic stirring for 1 min. This solution was then added to solution I. Solution II: An amount of 11.50 g of ammonium persulfate (APS) for PW_0.5_ and PW_1_ or 23 g for PW_2_ was dissolved in 200 mL of hydrochloric acid (HCl, 1 M). Solution II was then added, drop-by-drop, to solution I under constant magnetic stirring, allowing the aniline monomer polymerization and the obtainment of PW_0.5_, PW_1_ and PW_2_ nanocomposites.

### 3.2. X-ray Diffraction Measurements

The X-ray diffraction (XRD) measurements were performed on a Panalytical diffractometer, model Empyrean, K_α_Cu, operating at 50 kV and 100 mA. Measurements were performed from 2*θ* = 3°–100° with angular increment of 0.02° and 5 s/step.

### 3.3. FTIR and UV-VIS Spectroscopy

Fourier-transform infrared spectroscopy (FTIR) was performed on a Thermo Nicolet spectrophotometer, model NEXUS 470/FTIR, from 400 cm^−1^ to 4000 cm^−1^ and 64 scans. Ultraviolet-Visible (UV–VIS) measurements were performed on a Biotek Epoch 2 spectrophotometer from 200 nm to 800 nm.

### 3.4. Scanning Electron Microscopy (SEM)

Nanocomposite powder morphology was analyzed on a Supra 35 microscope, Carl Zeiss, using 1.0 kV. Powder samples were deposited on a carbon tape and coated with a thin gold layer. The surface morphology was obtained at 25 °C.

### 3.5. Complex Impedance Spectroscopy (CIS)

CIS measurements were performed on a Solartron 1260 impedance analyzer at 27 °C from 10^1^ Hz to 10^6^ Hz and 500 mV. Powdered samples were formed into pellets (12 mm in diameter; 2 mm in thickness) using an EZ-Press 12 Ton Hydraulic Press, and pressure of 6 ton for 15 min.

## 4. Theoretical and Computational Methods

The Density Functional Theory (DFT) plus the coulombic U interaction (DFT+U) [37] was performed in the program Quantum Espresso [34]. The functional of Perdew, Burke, and Ernzerhof (PBE) [38], based on the generalized gradient approximation (GGA), was used to describe the exchange-correlation energy. To perform the optimization geometry, the BFGS *quasi*-Newton algorithm of Broyden, Fletcher, Goldfarb e Shanno [39,40] was adopted, with convergence thresholds of 10^−3^ eV/A for force and 10^−4^ eV for energy. The van der Waals interaction was considered using the semi-empirical DFT-D2 method of Grimme [41] for a more accurately geometric optimization. The kinetic energy cutoff for the wave functions were 476 eV for the PANI structures and 612 eV for the WO_3_·2H_2_O phase and nanocomposite systems. A Monkhorst-Pack network was constructed in k space for the Brillouin zone, with dimension of (1 × 1 × 6) for PANI and (6 × 6 × 6) for WO_3_·2H_2_O and PWO nanocomposites. The structures’ graphical representations were obtained using the package XcrySDen [42]. The quantum transmittance and the electric current as a function of voltage were calculated using the package *Want* [33] implemented in the Quantum Espresso software [34]. Landauer’s formula [43] was used considering an infinite periodic system at low temperature. The Program Gaussian 03 [44] was applied to calculate the FTIR spectra using the PBE functional and a set of aug-cc-pvdz basis functions [45] forming the model (PBEPBE/aug-cc-pvdz).

## 5. Conclusions

Electronic and spectroscopic properties of nanocomposites formed by ES–PANI and WO_3_·2H_2_O were successfully evaluated, based on combined experimental characterization and theoretical calculations via DFT. We hope this paper may contribute to the preparation and characterization of conjugated polymer-based nanocomposites by adding metallic particles into the polymerization medium, allowing phase interaction and metal oxidation, as well as assist in understanding the interactions between the nanocomposite phases. Our results showed a novel synthesis methodology to prepare PANI–WO_3_·2H_2_O nanocomposite based on the oxidation of metallic W. After 2 h of synthesis, the resulting nanocomposite presented improved electrical conductivity when compared to the pure ES–PANI, accessing the mechanism of the electronic transitions by theoretical calculation. This increase in conductivity resulted from the electrostatic interactions between the polymer chains and WO_3_·2H_2_O, and the energy states of the polymer acted as an electron transfer bridge to the conduction states of the nanocomposite. A reduction of costs and in process steps may be achieved in the preparation of the ES–PANI and WO_3_·2H_2_O nanocomposite by this new alternative route of synthesis, when compared to its conventional synthesis, based on sodium tungstate di-hydrate.

## Figures and Tables

**Table 1:** Bond lengths and torsion angles of the PWO nanocomposites and their individual phases.

Torsion Angles (^o^)	Bond Lengths (Å)		
**ES–PANI ^†^**	**ES–PANI ^†^**	**WO_3_** **·2H_2_O ^†^**	
*Φ* = 26.43	d_1_ = 1.41	d_1_ = 2.32	d_6_ = 1.95
*φ* = 22.88	d_2_ = 1.37	d_2_ = 1.73	d_7_ = 1.00
*Ψ* = 29.76	d_3_ = 1.36	d_3_ = 1.90	d_8_ = 0.97
-	d_4_ = 1.06	d_4_ = 1.88	d_O12_ = 1.49
-	d_5_ = 1.80	d_5_ = 1.93	d_O34_ = 1.61
**ES–PANI ^‡^**	**ES–PANI ^‡^**	**WO_3_** **·2H_2_O ^‡^**	
*Φ* = 13.52	d_1_ = 1.42	d_1_ = 2.24	d_6_ = 1.90
*φ* = 04.03	d_2_ = 1.38	d_2_ = 1.72	d_7_ = 1.00
*Ψ* = 06.96	d_3_ = 1.36	d_3_ = 1.91	d_8_ = 0.94
-	d_4_ = 1.02	d_4_ = 1.88	d_O12_ = 1.17
-	d_5_ = 2.05	d_5_ = 1.91	d_O34_ = 1.18

^†^ Isolated structures; ^‡^ nanocomposite structures.

**Table 2:** Hydrogen bond lengths and distances between the oxide layers and polymer phase.

System	∆_1_ (Å)	∆_2_ (Å)	*d_Cl_*_−*H*_ (Å)	*d_O_*_−*H*_ (Å)
PWO–ClS	–	0.60	2.06 *	1.36–1.89
PWO–NS	–	0.44	–	1.40–2.02
PWO–ClB	1.17	1.78	2.36 **	1.46–1.82
PWO–NB	0.78	1.85	–	1.36–2.02

* Bottom oxide layer; ** upper oxide layer.

**Table 3:** Vibrational modes from the experimental FTIR spectrum of the nanocomposite phases.

Wavenumber (cm^−1^)	Vibrational Mode	Description
3218 ^†^	*υ*_s_ (N—H)	Symmetric stretching of the N–H bond
1560 ^†^	*υ*_s_ (N—Q—N)	Quinoid ring stretching
1472 ^†^	*υ*_s_ (N—B—N)	Benzenoid ring stretching
1295 ^†^, 1240 ^†^	*υ*_s_ (C—N^+^)	Stretching of the C–N^+^ bond of the bipolaron structure
1120 ^†^	*υ*_s_ (N—H^+^)	Stretching of the delocalized N–H^+^ bond referring to the delocalized *π*–electrons
800 ^†^	*γ* (C—H)	Out-of-plane deformation of C–H bonds ofbenzenoid rings
3420 *	*υ*_s_ (O—H)	Stretching of the O–H bond of water moleculespresent in the structure
1640 *	*δ* (O—H)	Angular deformation in the O–H plane
874 *, 814 *	*υ*_a_ (O—W—O)	Asymmetric stretching of O–W–O bonds
682 *, 590 *	*υ*_s_ (O—W—O)	Symmetric stretching of O–W–O bonds

^†^ Polymer phase; * Oxide phase.

**Table 4:** UV-VIS energy absorptions of the nanocomposites and the electronic transitions of their constituting phases.

Wavelength (nm)	Electronics Transitions
205	Transitions associated with pure tungsten
230	π–π* transitions of the benzenoid structure
282	π–π* transitions no-local
350	polaron–π* transitions
445	Transitions associated with tungsten oxide
840	*π–*polaron transitions

**Table 5:** Complex conductivity values.

Samples	$\sigma^{\prime }\;(S/\mathrm{cm})$		$\sigma^{\prime •}\;(S/\mathrm{cm})$	
	10 Hz	1 MHz	10 Hz	1 MHz
PW_0.5_	1.4 × 10^−1^	1.8 × 10^−1^	1.0 × 10^−3^	7.7 × 10^−3^
PW_1_	1.6 × 10^−2^	3.0 × 10^−2^	3.4 × 10^−4^	1.3 × 10^−2^
PW_2_	2.9 × 10^−2^	4.3 × 10^−2^	3.1 × 10^−4^	1.0 × 10^−2^

**Table 6:** Lowdin charge analysis between WO_3_·2H_2_O and the doped/undoped polymer phase, representing the direction of charge flow in PWO–ClS and PWO–NS nanocomposites.

System	Polymeric	Oxide
PWO–ClS	Δ*C_Lowdin_* (PANI–Cl)	Δ*C_Lowdin_* (WO_3_·2H_2_O)
	−0.2459	+0.2636
PWO–NS	Δ*C_Lowdin_* (PANI)	Δ*C_Lowdin_* (WO_3_·2H_2_O)
	−0.4961	+0.5226

## Data Availability

The data used to support the findings of this study are available from the corresponding author upon request.

## Sample Availability

Sample of the compounds are not available from the authors.
